# Glucagon-Like Peptide-1 Secreting L-Cells Coupled to Sensory Nerves Translate Microbial Signals to the Host Rat Nervous System

**DOI:** 10.3389/fncel.2020.00095

**Published:** 2020-04-30

**Authors:** Maria M. Buckley, Rebecca O’Brien, Eilish Brosnan, R. Paul Ross, Catherine Stanton, Julliette M. Buckley, Dervla O’Malley

**Affiliations:** ^1^Department of Physiology, University College Cork, Cork, Ireland; ^2^APC Microbiome Ireland, University College Cork, Cork, Ireland; ^3^College of Science, Engineering and Food Science, University College Cork, Cork, Ireland; ^4^Teagasc Food Research Centre, Cork, Ireland; ^5^Department of Surgery, University College Cork, Cork, Ireland; ^6^Mater Private Hospital, Cork, Ireland

**Keywords:** enteric neurons, indole, GLP-1, microbiota, vagus nerve, tryptophan

## Abstract

An intact gut epithelium preserves the immunological exclusion of “non-self” entities in the external environment of the gut lumen. Nonetheless, information flows continuously across this interface, with the host immune, endocrine, and neural systems all involved in monitoring the luminal environment of the gut. Both pathogenic and commensal gastrointestinal (GI) bacteria can modulate centrally-regulated behaviors and brain neurochemistry and, although the vagus nerve has been implicated in the microbiota-gut-brain signaling axis, the cellular and molecular machinery that facilitates this communication is unclear. Studies were carried out in healthy Sprague–Dawley rats to understand cross-barrier communication in the absence of disease. A novel colonic-nerve electrophysiological technique was used to examine gut-to-brain vagal signaling by bacterial products. Calcium imaging and immunofluorescent labeling were used to explore the activation of colonic submucosal neurons by bacterial products. The findings demonstrate that the neuromodulatory molecule, glucagon-like peptide-1 (GLP-1), secreted by colonic enteroendocrine L-cells in response to the bacterial metabolite, indole, stimulated colonic vagal afferent activity. At a local level indole modified the sensitivity of submucosal neurons to GLP-1. These findings elucidate a cellular mechanism by which sensory L-cells act as cross-barrier signal transducers between microbial products in the gut lumen and the host peripheral nervous system.

## Introduction

The peripheral nervous system innervating the colon has evolved in the continued presence of over a 100 trillion microbial organisms, mostly bacteria. These microbes are predominantly beneficial, scavenging additional calories, secreting vitamins and ensuring normal immune and gastrointestinal (GI) development, but it appears that they may also manipulate host physiology and behavior to their benefit (Stilling et al., 2016). Alterations in the luminal microbiota have been linked with stress-related disorders, Parkinson’s disease, autism spectrum disorder and schizophrenia (Dinan and Cryan, 2013, 2017). The vagus nerve has been implicated in the modulation of host behavior and altered central expression of neurotransmitters by putative probiotics (Bercik et al., 2011; Bravo et al., 2011; Perez-Burgos et al., 2013). Indeed, stimulation of vagal nerve activity (Perez-Burgos et al., 2013) and activation of intrinsic primary afferent neurons (Mao et al., 2013) in response to the exposure of mouse jejunum mucosa to *Lactobacillus Rhamnosus* JB-1 have been reported. Intrinsic primary afferent neurons may act as a neural starting point of gut-to-brain signaling (Perez-Burgos et al., 2014) and indeed, are less excitable in the absence of gut microbes (McVey Neufeld et al., 2013). However, a mechanistic understanding of how these bacterial signals are interpreted by the host is yet to be established.

**GRAPHICAL ABSTRACT:**
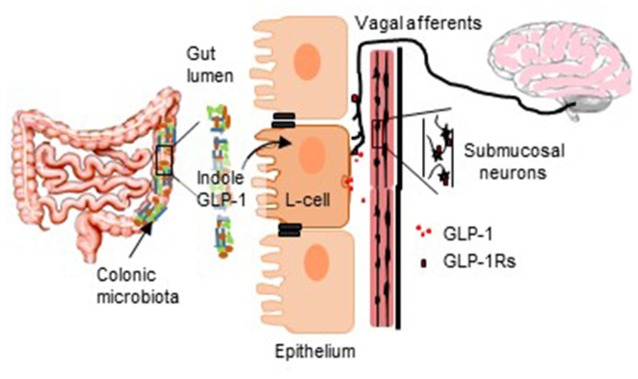
The diagram illustrates the proposed role of GLP-1-secreting L-cells in translating bacterially-originating signals to neurostimulatory actions.

Bacteria can infiltrate the gut (Pérez-Berezo et al., 2017; Jaglin et al., 2018) and, this is indeed more likely in disorders associated with increased GI permeability, such as Irritable Bowel Syndrome (IBS). However, given that the healthy gut is immunologically primed to detect and prevent bacterial penetration, it is likely that an integral homeostatic signaling mechanism, which maintains the integrity of the gut barrier, exists to facilitate microbiota-gut-brain signaling. Pathogen associated molecular patterns, which identify and evoke a host response to pathogenic microbes, are well described in the gut epithelium, and Nod-like receptors are implicated in gut-brain signaling (Pusceddu et al., 2019), however, other cells in the epithelium act as chemosensory transducers for non-threating gut stimuli. Serotonin biosynthesis was stimulated by chemical irritants, volatile fatty acid fermentation products and catecholamines (Yano et al., 2015), which subsequently modulated primary afferent nerve fibers *via* synaptic connections (Bellono et al., 2017). Thus, enterochromaffin cells transduce environmental, metabolic, and homeostatic information from the gut lumen to the nervous system. However, L-cells also act as biosensors of the gut lumen.

Electrically-excitable enteroendocrine L-cells are embedded in the epithelium and secrete glucagon-like peptide-1 (GLP-1) from their basolateral face following stimulation (Chimerel et al., 2014). L-cells are found throughout the small and large intestine (Hansen et al., 2013), but function differently depending on their location. For instance, small intestinal L-cells in humans (Sun et al., 2017) and rats (Kuhre et al., 2015) are sensitive to glucose, whereas *in vitro* colonic L-cells express bile receptors and receptors for short-chain fatty acids (Tolhurst et al., 2012). Bacterial metabolites such as indole (Chimerel et al., 2014), S-equol (Harada et al., 2018) and prebiotics (Gibson and Roberfroid, 1995; Cani et al., 2006) induce GLP-1 secretion, but conversely, GLP-1 is also elevated in germ-free mice (Wichmann et al., 2013). Although L-cells are classically described as endocrine cells, like enterochromaffin cells (Bellono et al., 2017), they can synapse directly with peripheral afferent and efferent neurons (Bohórquez et al., 2015), providing a direct neural pathway for bi-directional brain-gut communication (Kaelberer et al., 2018). Despite growing interest in the microbiota-gut-brain axis, relatively little is known about the chemosensory transduction of microbial signals across an intact barrier. In this study, we have investigated the capacity of L-cells to interpret bacterial signals from the gut lumen and activate host colonic afferents and intrinsic neurons by secreting GLP-1.

## Materials and Methods

### Ethical Approval

All animal experiments were in full accordance with the European Community Council Directive (86/609/EEC) and the local University College Cork Animal Experimentation Ethics Committee. Rats were sacrificed by CO_2_ overdose and perforation of the diaphragm.

### Animals and Tissue Collecting

Sprague–Dawley rats were used to determine if bacterial products could activate enteric neurons and the vagus nerve across an intact, non-leaky colon (Gareau et al., 2007). Male Sprague–Dawley rats (8–12 weeks) purchased from Envigo, Derbyshire, UK, were group-housed five per cage and maintained on a 12/12 h dark-light cycle (08.00–20.00) with a room temperature of 22 ± 1°C. Animals were permitted at least 1 week to acclimatize to their new environment before experimentation. Standard chow diet and water were available *ad libitum*. A section of colon 8 cm proximal to the anus was excised from each rat and maintained in ice-cold Krebs saline containing in mM/L: 117 NaCl, 4.8 KCl, 2.5 CaCl_2_, 1.2 MgCl_2,_ 25 NaHCO_3,_ 1.2 NaH_2_PO_4_ and 11 D-glucose (pH 7.4).

### Commensal Bacterial Strains

*Lactobacillus paracasei* NFBC 338 (*L.paracasei*) was transformed to secrete a long-acting analog of GLP-1, confirmed by mass spectrometry and *in vitro* assays of insulinotropic activity (Ryan et al., 2017). The engineered commensal bacteria were cultured at 1% (v/v) in de Man, Rogosa and Sharpe broth (Difco, VWR, Philadelphia, PA, USA) for ~17 h at 37°C under anerobic conditions [anerobic jars with Anaerocult A Gas Packs (Merck, Darmstadt, Germany)] until stationary phase and centrifuged (16,900× *g* for 15 min, at 4°C; SLA-3000 rotor, Sorvall RC B5-Plus). The cell pellet was washed twice with phosphate-buffered saline (PBS; Sigma Aldrich, UK), re-suspended at ~2 × 10^10^ CFU.ml^−1^ in sterile 15% trehalose (Sigma Aldrich), which acted as a cryoprotectant and 1 ml aliquots were dispensed into 2 ml lyophilization vials. The vials were lyophilized on a 24 h program (freeze temperature −40°C, additional freeze 1 min, condenser set point −60, vacuum setpoint 600 mTorr; VirTis AdVantage Wizard 2.0) and stored at 4 °C. Bacteria were resuspended in distilled water each day to deliver 1 × 10^10^ CFU.ml^−1^ for each exposure.

### Colonic-Afferent Nerve Electrophysiological Recordings

**Figure 1:**
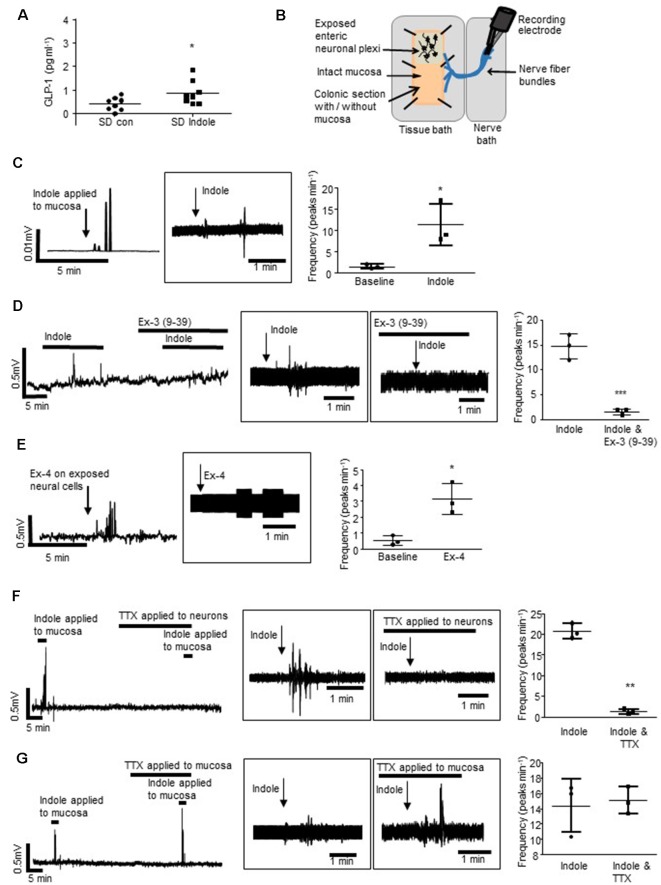
L-cells facilitate indole-evoked activation of vagal afferents.** (A)** The plot illustrates basolateral secretion of Glucagon-like peptide-1 (GLP-1) from colonic tissue apically exposed to indole (1 mM, 30 min) or saline (control). **(B)** The schematic (amended from Buckley and O’Malley, 2018) illustrates the *ex vivo* distal colonic tissue preparation. Recording is made from vagal afferents attached to the esophagus. The mucosa can be left intact, or removed to expose enteric neurons and associated afferent endings. **(C)** The rectified and raw (in boxes) traces show vagal nerve activity in response to stimulation of the distal colonic mucosa. Scatter plots illustrate peak frequency. Indole-evoked vagal nerve activity was attenuated by **(D)** exendin 3(9–39) amide [Ex-3(9–39)]. **(E)** Exendin-4 (Ex-4) stimulated vagal nerve firing when applied to the exposed neurons. **(F)** Tetrodotoxin (TTX) inhibited the indole-evoked response when it is applied to a hemisected piece of colonic tissue but not **(G)** colonic tissue with an intact mucosa. *p < 0.05, **p < 0.01, ***p < 0.001.

The detailed description of the *ex vivo* dissection technique and recording of distal colonic afferent nerves has been previously reported (Buckley and O’Malley, 2018). In brief, a segment of the esophagus with the attached posterior vagus nerve was excised from an adult Sprague–Dawley rat. Maintaining an intact neural connection to the esophagus, a segment of distal colon (5 cm from the anus) with attached inferior and superior mesenteric ganglia, celiac ganglia and vagus nerve were placed in a recording chamber. Adjacent Sylgard-lined chambers allowed the afferent nerves to be isolated from the distal colon. The colon was opened, mucosal side up and the vagus nerve was carefully threaded through to the adjacent chamber and the gap was sealed with petroleum jelly (Figure 1B). Both chambers were superfused with 5% CO_2_/95% O_2_ bubbled Krebs-buffered saline maintained at 37°C. Multi-unit neural activity was recorded using platinum bipolar recording electrodes (WPI, Sarasota, FL, USA) attached to a Power lab (AD Instruments, Oxford, UK). Reagents [indole (1 mM, Sigma-Aldrich: cat. No. I3408), exendin 3(9–39) amide (10 μM, Tocris; cat. No. 2081), exendin-4 (10 μM, Tocris: cat. No. 1933) or tetrodotoxin (10 nM, Tocris: cat. No. 1078)] were applied to the colonic bath in the superfusate. Nerve activity was viewed and analyzed with Chart 7 (AD Instruments, Oxford, UK). Changes in multi-unit neural activity in the vagal nerve are presented as frequency from raw traces. Both raw and rectified traces are presented.

### Immunofluorescence and Confocal Microscopy

Whole-mount colonic submucosal plexus (SMP) preparations were pinned out in Sylgard-lined dishes and fixed in 4% paraformaldehyde (4°C, overnight). The tissues were subsequently permeabilized with 0.1% Triton X-100 and blocked with 1% donkey serum (Sigma Aldrich). SMP tissue was incubated with primary antibodies (1:250 @ 4°C overnight) against GLP-1 [rabbit polyclonal antibody (ab22625), Abcam, Cambridge, UK (Duca et al., 2013)] or goat polyclonal antibody (sc7782; Santa Cruz Biotechnology Inc, TX, USA), GLP-1 receptors [GLP-1R, mouse polyclonal antibody (sc66911), Santa Cruz Biotechnology] and FITC-conjugated anti-rabbit or anti-goat and TRITC-conjugated anti-rabbit or anti-mouse secondary antibodies (1:250, 2 h at room temperature, Jackson Immunoresearch, Westgrove, PA, USA). Images were captured using an FVl0i-Olympus-confocal microscope with Fluoview software (FV10i-SW). No non-specific fluorescence was detected in control experiments where tissues were incubated with primary antibodies or secondary antibodies alone, or where anti-GLP-1R antibodies were neutralized with a blocking peptide before the staining protocol. Changes in the optical density of GLP-1R expression was quantified using ImageJ (National Institute of Health, USA). Membrane expression of GLP-1Rs from three neurons per ganglia in three different tissue preparations were compared when indole was applied to the submucosal neurons or the mucosa.

### Calcium Imaging

For calcium imaging studies, whole-mount preparations of SMP neurons were prepared from the distal colon of healthy Sprague–Dawley rats. The colon was mounted on a glass rod, where the outer serosal layer was scored lightly with a blade along the mesenteric border. To prepare an SMP tissue preparation, the serosal and mucosal layers were removed and the colonic tissue (~2 cm × 2 cm) was pinned out in Sylgard-lined dishes. Submucosal neuronal preparations were loaded with Fura-2AM (7 μM, 1 h, Thermo-Fisher Scientific, UK) or Fluo-8 (8 μM, 1 h, Abcam, UK) in Krebs-buffered saline solution comprised of (in mM l^−1^): NaCl, 117; KCl, 4.8; CaCl_2_, 2.5; MgCl_2_, 1.2; NaHCO_3_, 25; NaH_2_PO_4_, 1.2 and D-glucose, 11; prior to imaging. SMP tissue preparations were used to investigate the activation of submucosal neurons in the absence of epithelial and enteroendocrine cells and their secretions. A hemisected colonic tissue preparation (Mao et al., 2013), where the SMP is exposed on one half of the tissue but the mucosa is left intact on the other half (Figure 3E), was used to compare calcium responses evoked by neuronal exposure to Ex-4 before and after mucosal application of indole. The presence of the epithelium indicates that mucosally-secreted factors are implicated in the modification of neuronal function. Reagents [indole (1 mM), exendin-4 (10 μM), exendin 3(9–39) amide (10 μM), thapsigargin (100 nM, Tocris: cat. No. 1138), ω-agatoxin IVA (100 nM, Sigma Aldrich: cat. No. A6719), ω-conotoxin GVIA (100 nM, Sigma Aldrich: cat. No. C9915), wortmannin (100 nM, Sigma Aldrich: cat. No. W1628), WP1006 (1 μM, Calbiochem) and PD98059 (1 μM, Sigma Aldrich: cat. No. 513000)] were added to the perfusate. Images were acquired at 1 Hz using a conventional fluorescence imaging system (Cairn Life technologies, UK or Olympus, Melville, NY, USA), and a water-immersion objective (Olympus, 40× magnification, numerical aperture: 0.80) on a fixed stage upright microscope (Olympus BX51WI). Cell R software (Olympus Soft imaging solutions, Munster, Germany) or WinFluor Fluorescence Image Capture and Analysis program (John Dempster, University of Strathclyde, Scotland) was used to record excitation changes in intracellular calcium (O’Malley et al., 2011b).

**Figure 2:**
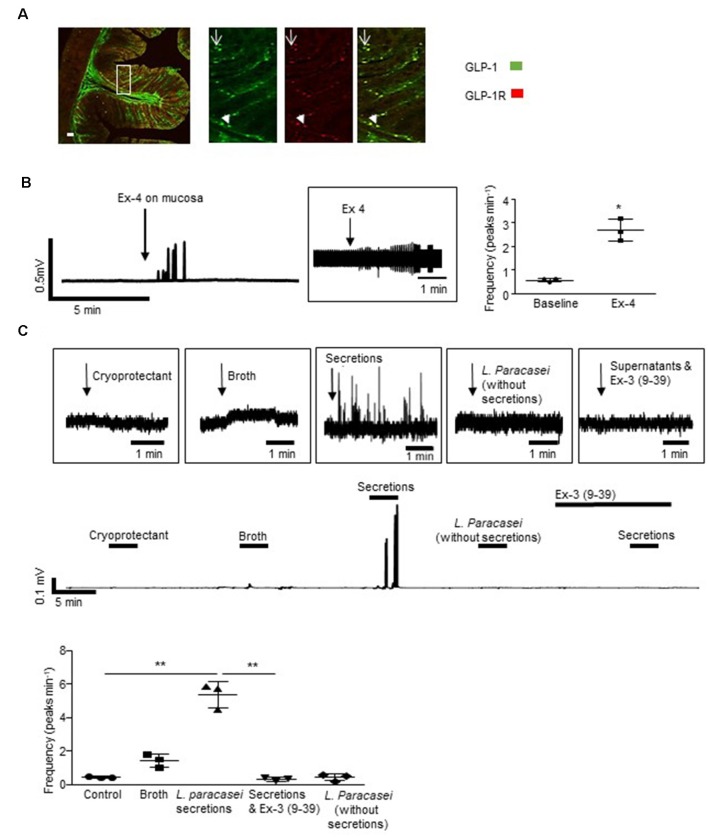
*Lactobacillus paracasei* secretions stimulate host neurons. **(A)** The representative immunofluorescent image of a rat colonic cross-section illustrates that some L-cells expressed GLP-1Rs (arrowhead) but others did not (arrow). **(B)** Mucosal application of exendin-4 (Ex-4) increased vagal activity. **(C)** Increased vagal nerve activity evoked by *L. paracasei* secretions was abolished by Ex-3(9–39). *p < 0.05, **p < 0.01. Scale bars: 50 μm.

**Figure 3:**
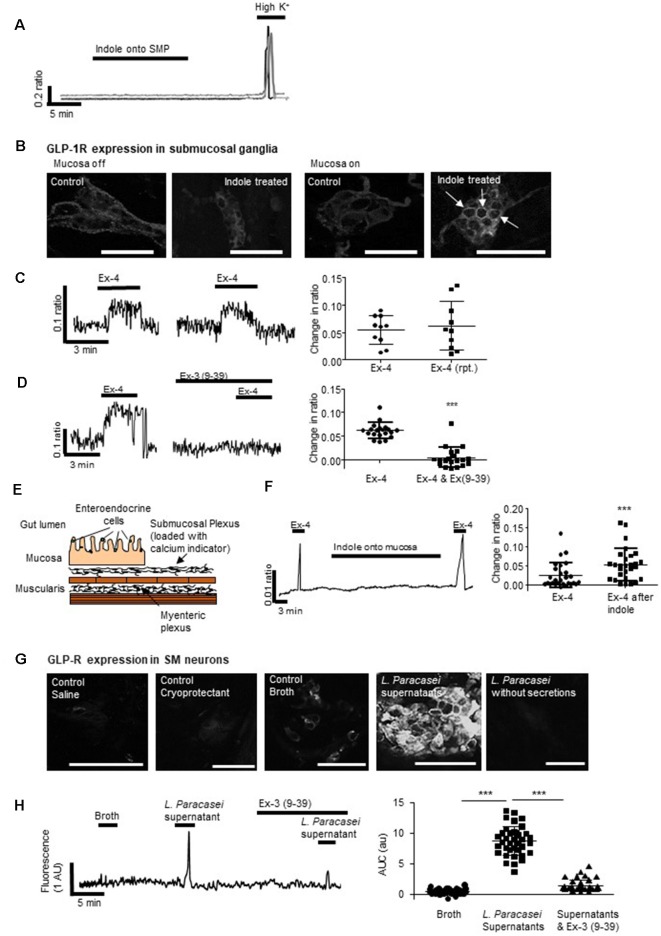
Mucosal L-cells facilitate indole-evoked activation of submucosal neurons. **(A)** Indole did not affect intracellular calcium ([Ca^2+^]_i_) levels in submucosal neurons. **(B)** Immunofluorescence images show the GLP-1 receptor (GLP-1R) expression in colonic submucosal neurons following neuronal and mucosal exposure to indole. Arrows indicate increased expression at the neuronal cell membranes. Scalebar: 50 μm. **(C)** The GLP-1R agonist, exendin 4 (Ex-4) stimulated an increase in [Ca^2+^]_i_, a reproducible effect that was **(D)** attenuated by the GLP-1R antagonist, exendin 3(9–39) amide [Ex-3(9–39)]. **(E)** The schematic illustrates a cross-section of the hemisected distal colon. By removing half of the mucosa, changes in intracellular calcium can be recorded from submucosal neurons loaded with a calcium indicator dye. By leaving some of the mucosa intact, the role of endocrine hormones secreted by epithelial cells can be investigated. **(F)** In hemisected colonic tissue, the Ex-4-evoked calcium response was enhanced by mucosal indole.** (G)** Immunofluorescent images show GLP-1R expression in colonic submucosal neurons following mucosal stimulation with saline, cryoprotectant, broth, *L. paracasei* secretions, and *L. paracasei* bacteria. Scalebar: 50 μm. **(H)** Mucosal application of *L. paracasei* secretions evoked a calcium response in submucosal neurons which was attenuated by the GLP-1R antagonist, exendin 3(9–39) amide [Ex-3(9–39)]. ***p < 0.001.

As previously described (O’Malley et al., 2011a), submucosal neurons were identified based on morphology and responsivity to brief application of 75 mM KCl at the end of the recording protocol. Reagents were applied in a random order to avoid the run-down of calcium stores being a confounding factor. The colonic tissue was continuously superfused with carbogen-bubbled Krebs-buffered saline at room temperature containing the L-type calcium channel blocker, nifedipine (1 μM, Sigma-Aldrich, cat. No.: N7634), to inhibit smooth muscle contraction. Responding neurons were defined as those which exhibited an increase in intracellular calcium ([Ca^2+^]_i_) that was greater than two standard deviations from the baseline values in that cell (calculated during the 150 s preceding stimulus application). Paired analysis of responses within a single neuron were compared to determine the effect of the pharmacological reagent on the control response.

### Ussing Chamber Electrophysiology

Mucosa-submucosal preparations of distal colon were mounted in Ussing chambers (exposed area of 0.12cm^2^) with 5 mls of Krebs saline solution (95% O_2_/5% CO_2_, 37°C) in the basolateral and luminal reservoirs. Tissues were voltage-clamped at 0 mV using an automatic voltage clamp (EVC 4000, World Precision Instruments, Sarasota, FL, USA) and the short-circuit current (I_SC_) required to maintain the 0 mV potential was monitored as a recording of the net active ion transport across the epithelium. Experiments were carried out simultaneously in all chambers and connected to a PC equipped with DataTrax II software (WPI). This software was used to measure the peak response and resistance was calculated using Ohms law. Following mounting, tissue was allowed to equilibrate (~1 h). Reagents [exendin 4 (10 μM), carbachol (10 μM, Sigma-Aldrich, cat. No.: Y0000113), veratridine (10 μM, Sigma-Aldrich, cat. No.: V5754), capsaicin (1 μM, Sigma-Aldrich, cat. No.: M2028)] were added to the basolateral chamber.

Separation of the basolateral and apical surfaces in the Ussing chambers were exploited to determine if basolateral secretion of GLP-1 by colonic L-cells was induced by indole. Distal colonic tissue was mounted in low-volume (1 ml carbogenated Krebs saline solution) Ussing chambers with an exposed tissue area of 0.64 cm^2^. Following mucosal exposure to indole (1 mM, 30 min), secretion of GLP-1 into the basolateral reservoir (1 ml) was ascertained by immunoassay.

### Mesoscale Discovery Biomarker Assay

An immunoassay (MesoScale Discovery U-PLEX customized multiplex assay kit I, MesoScale Discovery, Gaithersburg, MD, USA) with a dynamic range for GLP-1 of 0.02–120 pM, was carried out to determine if GLP-1 was secreted in response to indole as compared to saline-treated controls. The assay was run in triplicate and an electrochemiluminescent detection method was used to measure protein levels in each sample. The plates were read using the MesoScale Discovery plate-reader (MESO QuickPlex SQ 120). A calibration curve was generated using standards, and GLP-1 concentrations were determined from the curve.

### Statistical Analyses

Data were analyzed using GraphPad prism for windows (version 5, Graphpad Software, San Diego, CA, USA). The data are represented as data plots with mean ± the standard deviation. Paired or unpaired *t*-tests or repeated-measures ANOVA with Tukey multiple comparison *post hoc* test, as appropriate, were used to compare data. *P* ≤ 0.05 was considered significant.

## Results

### Indole Indirectly Stimulates Activity in Colonic Vagal Afferents

Sprague–Dawley rats were used to determine if indole, a bacterial metabolite of tryptophan, which stimulates L-cells (Chimerel et al., 2014), can induce cross-barrier signaling in healthy, non-leaky colons (Gareau et al., 2007). In Ussing chamber experiments, exposure of the intact colonic apical epithelial surface to indole (1 mM, 30 min) resulted in secretion of GLP-1 into the basolateral reservoir as compared to saline-treated control tissues (*n* = 8 rats, *p* = 0.0355, Figure 1A). To investigate if bacterial products may use L-cells to activate the gut-brain neural signaling axis, an *ex vivo* preparation of rat distal colon with intact colonic afferents was used (Figure 1B; Buckley and O’Malley, 2018). Consistent with previous reports (Richards et al., 1996), baseline afferent activity was low. However, mucosal application of indole stimulated a robust increase in vagal nerve activity (*n* = 3 rats, *p* = 0.0264, Figure 1C). Incubation of the tissue with the GLP-1R antagonist, exendin-3 (9–39) amide [Ex-3 (9–39)], attenuated the indole-evoked response (*n* = 4, *p* = 0.001, Figure 1D). Given that GLP-1Rs are expressed in the nodose ganglia (Nakagawa et al., 2004), where cell bodies of afferent vagal neurons are located, basolaterally released GLP-1 could activate vagal afferents. Indeed, direct application of Ex-4 to a tissue preparation with exposed colonic submucosal neurons and afferent nerve endings similarly resulted in increased vagal firing (*n* = 3, *p* = 0.0219, Figure 1E), although in the absence of single-unit recordings it is not possible to determine if the same fibers are activated by both indole and Ex-4. In a hemisected colonic tissue preparation (Mao et al., 2013), where submucosal neurons and afferent and efferent nerve endings are exposed, indole-evoked stimulation of vagal nerves was blocked by the neurotoxin, tetrodotoxin (*n* = 3, *p* = 0.0047, Figure 1F). Interestingly, in colonic tissue sections where the epithelial layer was left *in situ*, thereby segregating intrinsic and extrinsic neurons from the luminally-applied tetrodotoxin, indole-evoked vagal stimulation was not inhibited (*n* = 3, *p* = 0.6821, Figure 1G). Thus, action potential generation is crucial for afferent signaling evoked by indole. However, stimulation of neural firing is initiated on the basolateral side of the epithelium. This is consistent with the basolateral secretion of GLP-1 in response to luminal application of indole.

### GLP-1 Secreted by *L. paracasei* Stimulates Colonic Afferents

Some, but not all GLP-1 immuno-labeled L-cells in the colonic epithelium of SD rats (*n* = 4 rats, Figure 2A) expressed GLP-1Rs. Thus, GLP-1 originating from sources in the gut lumen could directly or indirectly activate L-cells. Indeed, mucosal application of the GLP-1R agonist, exendin-4 (Ex-4, 10 μM) increased vagal firing (Figure 2B, *n* = 3, *p* = 0.0124). Although a native GLP-1-secreting microbe has yet to be characterized, a genetically recombineered *Lactobacillus paracasei* NFBC 338 (*L. paracasei*), which secretes a long-lasting analog of GLP-1, has been shown to signal across the gut to modify metabolic physiology (Ryan et al., 2017). To demonstrate that luminal application of a bacterial product can modify neural signaling, *L. paracasei* secretions were applied to the mucosa and similarly evoked vagal activation (*p* = 0.0074), an effect that was abolished by the GLP-1R antagonist, Ex-3 (9–39; *n* = 4 rats, *p* = 0.0095, Figure 2C). Application of control solutions (bacterial cryoprotectant, culture broth or the probiotics in the absence of their secretory products) did not affect vagal firing.

### Indole Indirectly Stimulates Colonic Submucosal Neurons Through the Activation of GLP-1 Receptors (GLP-1Rs)

Indole can penetrate the gut barrier (Jaglin et al., 2018), however, our studies found that even if this microbial product did cross the epithelium, cytosolic intracellular calcium ([Ca^2+^]_i_) in underlying submucosal neurons, important neural regulators of GI absorpto-secretory function, was unchanged in its presence (1 mM, *n* = 32 neurons from three SMP preparations, Figure 3A). Ganglionic expression of GLP-1Rs, which are most evident in neuronal cell membranes and extra-neuronal cells, was unchanged by direct exposure of submucosal neurons to indole (optical density: 11.3 ± 1.0 vs. 10.9 ± 1.2, *n* = 9 neurons from three tissue preparations, *p* = 0.804, Figure 3B). In contrast, application of indole to colonic tissue with an intact epithelium resulted in increased GLP-1R expression (optical density: 7.6 ± 0.3 vs. 23.4 ± 3.3, *n* = 9 neurons from three tissue preparations, *p* = 0.0002, Figure 3B) in submucosal ganglia, which is particularly evident at the cell membranes (indicated by arrows).

To emulate local paracrine actions of basolaterally-secreted GLP-1 on submucosal neurons, a colonic SMP preparation was exposed to Ex-4, a GLP-1 mimetic. Forty-four percentage (*n* = 14/34 neurons from three SMP preparations) of neurons responded to Ex-4 with a robust, but variable, increase in [Ca^2+^]_i_. The calcium response was reproducible upon second application (*n* = 10 neurons from three SMP preparations, *p* = 0.489, Figure 3C) and, consistent with previous studies in myenteric neurons (O’Brien et al., 2019), Ex-3 (9–39) abolished this response (*n* = 22 neurons from three SMP preparations, *p* < 0.0001, Figure 3D). In hemisected colonic tissue, where half of the mucosa was retained (Figure 3E), the mucosal application of indole did not affect neuronal calcium levels *per se* (Figure 3F). However, the neuronal calcium response evoked by Ex-4 (*n* = 29 from three SMP preparations, Figure 3F) was potentiated both in duration and amplitude (*p* < 0.0001) following exposure of the colonic mucosa to indole.

Expression of GLP-1Rs was increased in submucosal neurons following mucosal application of *L. paracasei* secretions but not broth (optical density:14.1 ± 1.4 vs. 29.5 ± 2.0, *n* = 9 neurons from three tissue preparations, *p* < 0.0001). This was primarily at neuronal cell membranes (Figure 3G). Mucosal application of *L. paracasei* secretions to hemisected colonic tissue increased [Ca^2+^]_i_ in submucosal neurons (*p* < 0.0001), a response that was attenuated by Ex-3(9–39; *n* = 38 neurons from three SMP preparations, *p* < 0.0001, Figure 3H).

### Exendin-4 Induces Calcium Influx From Extracellular Sources in Submucosal Neurons

**Figure 4:**
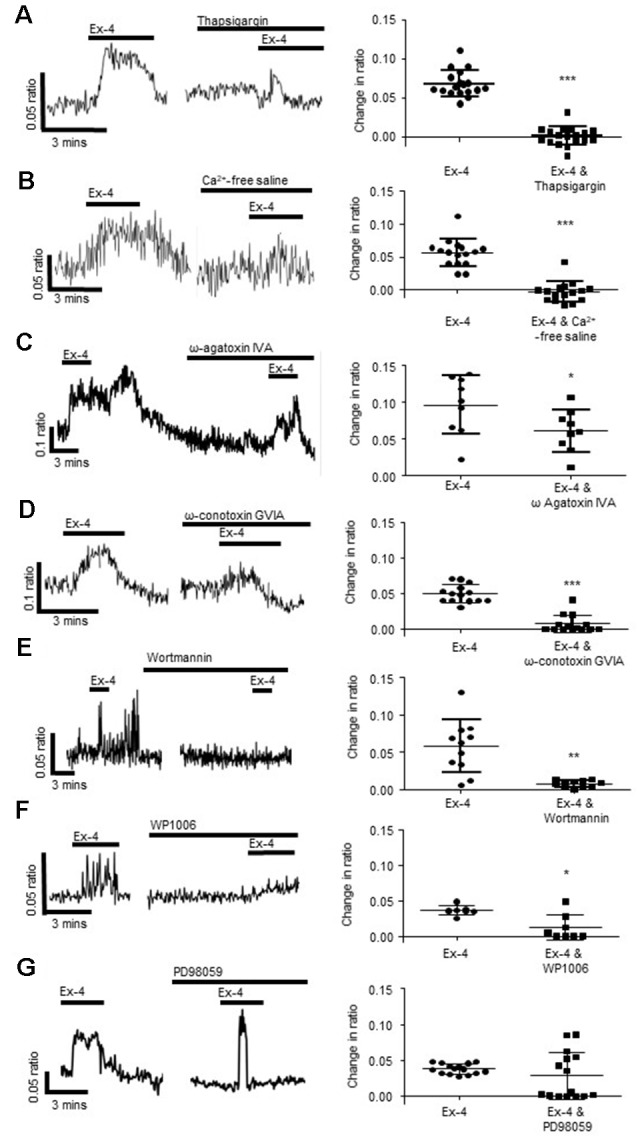
Exendin-4 stimulates calcium release from intracellular stores and influx *via* voltage-gated calcium channels.** (A)** The calcium response evoked by exendin-4 (Ex-4, 10 μM) was attenuated by thapsigargin (100 nM, *n* = 35) and **(B)** removal of extracellular calcium (*n* = 17). Specific inhibitors of **(C)** P/Q-(ω agatoxin IVA 100 nM, *n* = 9) and **(D)** N-(ω-conotoxin GVIA, 100 nM, *n* = 14) type voltage gated calcium channels inhibited Ex-4-evoked calcium responses. **(E)** The phosphoinositide 3-kinase (PI 3-kinase) inhibitor, wortmannin (100 nM, *n* = 11), and **(F)** the STAT3 inhibitor, WP1006 (1 μM, *n* = 8) attenuated Ex-4-evoked calcium responses. **(G)** The ERK-MAPK inhibitor, PD98059 (1 μM, *n* = 15), had no effect. *p < 0.05, **p < 0.01, ***p < 0.001.

To understand the cellular mechanisms evoked by activation of neuronal GLP-1Rs after indole-evoked GLP-1 release from colonic L-cells, the neuromodulatory actions of the GLP-1R agonist, Ex-4 (3-min application) was characterized further in the submucosal neurons. Our studies found that the Sarco/endoplasmic reticulum Ca^2+^ ATPase inhibitor, thapsigargin (100 nM, 30 min), reduced but did not abolish calcium responses evoked by Ex-4 (*n* = 35 neurons from three SMP preparations, *p* < 0.0001, Figure 4A), implicating calcium release from intracellular stores in submucosal neurons. However, removal of extracellular calcium also attenuated the Ex-4-evoked response (*n* = 16 neurons from three SMP preparations, *p* < 0.0001, Figure 4B) indicating the importance of the extracellular influx of calcium. Indeed, the P/Q-channel blocker, ω-agatoxin IVA (100 nM, 30 min, *n* = 9 neurons from three SMP preparations, *p* = 0.032, Figure 4C), and the N-channel blocker, ω-conotoxin GVIA (100 nM, 30 min, *n* = 14 neurons from three SMP preparations, *p* < 0.0001, Figure 4D), attenuated the calcium response evoked by Ex-4. To inhibit smooth muscle contraction, all calcium imaging studies were carried out in the presence of nifedipine (1 μM), an L-type calcium channel blocker, which did not impact on the capacity of Ex-4 to stimulate a calcium response.

Further studies were carried out to understand the intracellular signaling cascades evoked by Ex-4 in submucosal neurons. The Ex-4-evoked calcium response was attenuated by the phosphoinositide 3-kinase (PI 3-kinase) inhibitor, wortmannin (100 nM, 30 min; *n* = 11 neurons from three SMP preparations, *p* = 0.0013, Figure 4E) and the Signal Transducer and Activator of Transcription (STAT)-3 inhibitor, WP1006 (1 μM, 30 min, *n* = 8 neurons from three SMP preparations, *p* = 0.0102, Figure 4F), while the extracellular-signal-regulated kinase, mitogen-activated protein kinase (ERK-MAPK) inhibitor, PD98059 (1 μM, 30 min) had no effect on the Ex-4 evoked increase in [Ca^2+^]_i_ (*n* = 15 neurons from three SMP preparations, *p* = 0.2295, Figure 4G).

### The GLP-1R Agonist, Exendin-4 Modifies Secretory Currents and Gut Permeability

**Figure 5:**
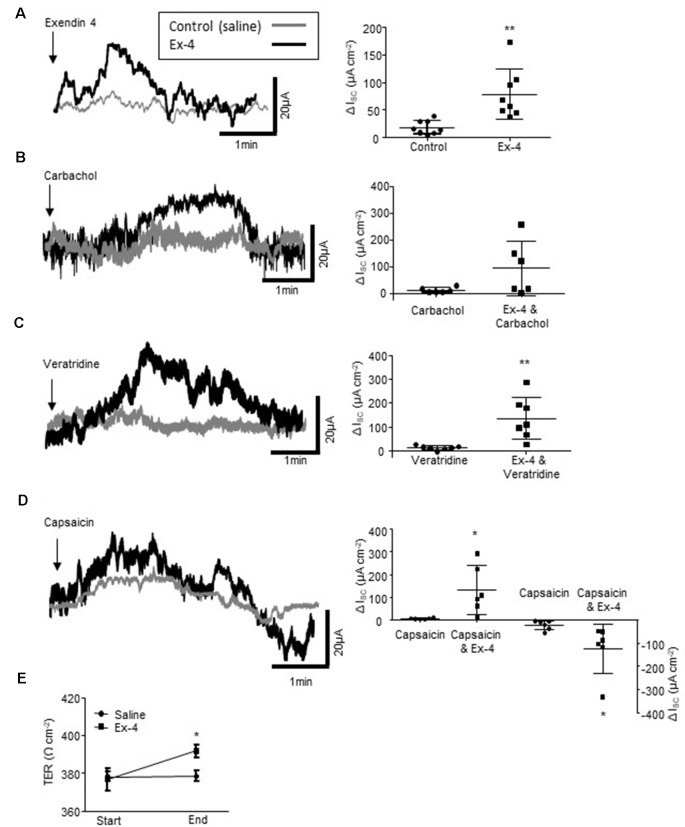
Exendin-4 enhances colonic secretory currents.** (A)** The representative secretory traces from Ussing chamber studies and the associated scatter plots illustrate the secretory current evoked by the GLP-1R agonist, exendin 4 (Ex-4, 10 μM, *n* = 8). **(B)** Ex-4 does not modify the cholinergically-mediated secretory current (I_SC_) evoked by carbachol (10 μM, *n* = 8) but enhances currents evoked by the **(C)** sodium channel agonist, veratridine (10 μM, *n* = 8) and **(D)** the sensory nerve stimulant, capsaicin (1 μM, *n* = 8). **(E)** Ex-4 also increases transepithelial resistance (TER) throughout an experiment (60–90 min). *p < 0.05, **p < 0.01.

As Ex-4 modifies the excitability of the SMP and this is the neural regulator of gut absorpto-secretory function, Ussing chambers were used to examine the impact of the GLP-1R agonist on colonic absorpto-secretory currents. As GLP-1 is secreted from the basolateral face of L-cells (Figure 1A), Ex-4 was added to the basolateral chamber of the Ussing chambers. Incubation with Ex-4 resulted in a small secretory current (*n* = 8 rats, *p* = 0.0052, Figure 5A). Whilst, Ex-4 did not modify secretory currents evoked by the cholinergic agonist, carbachol (*n* = 6, *p* = 0.0815, Figure 5B), currents evoked by the Na^+^ channel agonist, veratridine were potentiated (*n* = 8, *p* = 0.0085, Figure 5C). The afferent nerve stimulant, capsaicin evokes a biphasic secretory and anti-secretory response in colonic tissue. Ex-4 enhanced both the initial secretory phase (*n* = 6, *p* = 0.0156) and the anti-secretory response (*p* = 0.0462, Figure 5D). Moreover, the relative change in transepithelial resistance (TER), which is a measure of gut leakiness, throughout the experiment (60–90 min), increased in tissues continuously exposed to Ex-4 (*n* = 8, *p* < 0.05) compared to the saline-treated control preparations, which showed no change (Figure 5E). This finding suggests that the sustained presence of a GLP-1R agonist reduces colonic permeability.

## Discussion

An intact gut epithelium preserves the immunological exclusion of “non-self” entities in the external environment of the gut lumen. However, despite this physiological defense mechanism, information flows continuously across this interface. Enteroendocrine cells sense stimuli such as nutrients, inflammatory products, irritants, and microbial factors and respond by releasing hormones on the basolateral side of the gut barrier to evoke physiological responses (Gribble and Reimann, 2016). Some sensory epithelial cells, such as serotonin-secreting enterochromaffin cells (Bellono et al., 2017) and GLP-1-secreting L-cells (Bohórquez et al., 2015) form direct neural connections with afferent and efferent nerve fibers, meaning they are well equipped to translate luminal signals across an intact gut barrier to the host nervous system. In this study, we have investigated the potential of GLP-1 as a cross-barrier signaling molecule with stimulatory actions both on colonic afferents signaling to the CNS and locally, on submucosal neurons. The neurostimulatory actions of the bacterial metabolite, indole and GLP-1-secreting *L. paracasei* from the luminal side of the colon have been observed. Moreover, we have noted the need for an intact epithelium. Combined with the role of neuronal GLP-1R activation, our data implicates L-cells as cellular transducers that couple microbial signaling molecules with activation of the host peripheral nervous system.

Acute exposure to indole stimulated basolateral secretion of GLP-1 from rat colonic tissue, which is likely to be mediated through direct activation of L-cells by indole (Chimerel et al., 2014) but could also be through indirect mechanisms. Indeed, indole can modify epithelial cells and tight junction proteins (Bansal et al., 2010). Consistent with the importance of the vagus in informing the CNS about changes in the gut microbiome (Bercik et al., 2011; Bravo et al., 2011; Riley et al., 2013), electrophysiological studies demonstrated that application of indole to the mucosa, but not directly onto exposed intrinsic or extrinsic neurons, resulted in GLP-1R-dependent activation of vagal afferents. In terms of gut-brain axis wiring, most innervation of small intestinal epithelial cells is by submucosal neurons (Keast et al., 1984; Ekblad et al., 1987) and intrinsic primary afferent neurons may act as the starting point in gut-brain signaling (Perez-Burgos et al., 2014). Interestingly, longer exposure times suppress GLP-1 release (Chimerel et al., 2014), which may be important in gut-brain signaling regulation.

Mechanistically, inhibition of indole-evoked stimulation of vagal afferents in hemisected SMP tissue with tetrodotoxin indicates the role of action potentials in signal generation. However, when the epithelial layer was left intact, tetrodotoxin did not modify vagal activation by indole. This is consistent with nerve endings terminating below the epithelium and further implicates cells embedded in the epithelium in cross-barrier signaling. We also found that mucosally-applied Ex-4 and GLP-1-secreting *L. paracasei* evoked vagal firing in colonic tissue with an intact epithelium, which may be mediated through activation of GLP-1Rs on L-cells leading to basolateral secretion of GLP-1 and subsequent activation of vagal afferents (Bohórquez et al., 2015). Indeed, the effects of *L. paracasei*, which was applied to colonic sections with intact mucosa, were attenuated by the GLP-1R antagonist.

Application of indole to submucosal neurons, which lie in closest proximity to the mucosal layer and regulate absorpto-secretory function in the gut, had no impact on neuronal calcium levels in the absence of the epithelium. However, in contrast to the indirect mechanism underlying indole-evoked activation of the vagus nerve, submucosal neurons in hemisected tissue preparations were not activated by indole either, although we acknowledge that carrying out these studies at room temperature may have had an impact on temperature-sensitive elements. Nonetheless, the mucosal application of indole did enhance both the amplitude and duration of Ex-4-evoked calcium responses. This change in intracellular calcium levels could reflect slow excitatory postsynaptic potentials, slow inhibitory postsynaptic potentials or inhibitory slow after hyperpolarization seen in intrinsic primary afferent neurons, which are all dependent upon changes in intracellular calcium. Further studies will be needed to specifically determine which neuronal subtypes are affected. GLP-1R expression was increased in submucosal ganglia, which given the brief timeframe is likely to be due to the trafficking of GLP-1Rs to the cell membrane (Jones et al., 2018). Comparable to the neurostimulatory effects of GLP-1 in the nodose ganglia (Kakei et al., 2002) and central neurons (Ma et al., 2007), Ex-4 stimulated a robust increase in [Ca^2+^]_i_ in submucosal neurons. This was dependent upon activation of GLP-1Rs, which we and others (Kedees et al., 2013) have detected on submucosal neurons. Mucosal application of the secretory products of GLP-1-secreting *L. paracasei* similarly resulted in increased membrane expression of GLP-1R immunolabeling in submucosal ganglia, and evoked a GLP-1R-dependent calcium response. Although the ability of this engineered bacteria to modulate host physiology has been reported (Ryan et al., 2017), the mechanisms of action had not previously been elucidated. L-cells are reported to express a variety of receptors (Reimann et al., 2008; Tolhurst et al., 2012) but a study in mice indicated they do not express GLP-1Rs (Grigoryan et al., 2012). By contrast, we detected that some, but not all GLP-1-immuno-labeled L-cells in rat colon expressed GLP-1Rs. Thus, GLP-1 secreted luminally by *L. paracasei* could stimulate the basolateral release of GLP-1 from L-cells in an autocrine manner, which in turn could activate intrinsic and extrinsic neurons. Pharmacological characterization of the Ex-4-evoked calcium response revealed that both, calcium release from intracellular stores and influx from extracellular sources, contributes to the response. The downstream signaling molecule, PI 3-kinase appears to play a role in Ex-4-induced activation of submucosal neurons and the STAT3 inhibitor attenuated, but did not abolish, the calcium response.

In terms of the known consequences of the neurostimulatory actions of the GLP-1 agonist on GI function, GLP-1 suppresses GI contractile activity (Schirra et al., 2006; Hellström et al., 2008), but less was known about the actions of GLP-1 on intestinal secretory activity (Baldassano et al., 2012). Bearing in mind potential confounders of *ex vivo* experiments, including a lack of circulation and the possible deterioration of tissue integrity, we demonstrated that Ex-4 induced a secretory current and enhanced secretion evoked by the voltage-sensitive Na^+^ channel agonist, veratridine and capsaicin, which is known to activate vagal afferents (Blackshaw et al., 2000); findings that are consistent with the stimulatory effects of Ex-4 on submucosal neurons. Similar to a previous study (Baldassano et al., 2012), we did not find that GLP-1 impacted on carbachol-evoked secretory responses. However, consistent with our finding that indole enhanced the amplitude and duration of Ex-4-evoked calcium responses in submucosal neurons, microbial-sensing by L-cells may result in enhancement of neurally-regulated colonic secretion. Indeed, acute application of indole prolongs GLP-1 secretion from L-cells (Chimerel et al., 2014). Ex-4 increased colonic TER, indicating that the gut was less permeable. This may reflect a local protective response to prevent penetration of the gut by bacterial products and contribute to the anti-inflammatory actions of Ex-4 in the intestine (Kissow et al., 2013).

## Conclusion

These studies implicate GLP-1 as a signaling molecule in cross-barrier communication between luminal bacteria and the host peripheral and central nervous systems. Strengths of our experimental design include the ability to record nerve fiber activity signaling to the CNS from the distal colon in real-time, although a lack of pre-planned power calculations for these studies is a design limitation. Understanding this mechanism may be important in appreciating the pathophysiology of several diseases, particularly those associated with microbial dysbiosis. Indeed, in diarrhea-predominant IBS, tryptophan, the precursor of indole is elevated (Christmas et al., 2010). Moreover, increased numbers of afferent neurons expressing GLP-1Rs have been reported in inflammatory bowel disease, which may be important in relaying visceral pain signals in the diseased gut (Anand et al., 2018). Thus, microbial sensing by L-cells and subsequent activation of colonic afferents and local enteric neurons by GLP-1 may play a key role in neural dysregulation of gut function.

## Data Availability Statement

The datasets generated for this study are available on request to the corresponding author.

## Ethics Statement

The animal study was reviewed and approved by University College Cork Animal Experimentation Ethics Committee.

## Author Contributions

MB, RO’B, and EB: generated and analyzed data. RR and CS: provided probiotics. JB: tissue samples. DO’M: generated and analyzed data, supervised study, drafted the manuscript.

## Conflict of Interest

The authors declare that the research was conducted in the absence of any commercial or financial relationships that could be construed as a potential conflict of interest.

## References

[B1] Anand U. Yiangou Y. Akbar A. Quick T. MacQuillan A. Fox M.. (2018). Glucagon-like peptide 1 receptor (GLP-1R) expression by nerve fibres in inflammatory bowel disease and functional effects in cultured neurons. PLoS One 13:e0198024. 10.1371/journal.pone.0198024 29813107 PMC5973579

[B2] Baldassano S. Wang G. D. Mulè F. Wood J. D. (2012). Glucagon-like peptide-1 modulates neurally-evoked mucosal chloride secretion in guinea pig small intestine *in vitro*. Am. J. Physiol. Gastrointest. Liver Physiol. 302, G352–G358. 10.1152/ajpgi.00333.2011 22075777 PMC3287398

[B3] Bansal T. Alaniz R. C. Wood T. K. Jayaraman A. (2010). The bacterial signal indole increases epithelial-cell tight-junction resistance and attenuates indicators of inflammation. Proc. Natl. Acad. Sci. U S A 107, 228–233. 10.1073/pnas.0906112107 19966295 PMC2806735

[B4] Bellono N. W. Bayrer J. R. Leitch D. B. Castro J. Zhang C. O’Donnell T. A.. (2017). Enterochromaffin cells are gut chemosensors that couple to sensory neural pathways. Cell 170, 185.e16–198.e16. 10.1016/j.cell.2017.05.034 28648659 PMC5839326

[B5] Bercik P. Park A. J. Sinclair D. Khoshdel A. Lu J. Huang X.. (2011). The anxiolytic effect of Bifidobacterium longum NCC3001 involves vagal pathways for gut-brain communication. Neurogastroenterol. Motil. 23, 1132–1139. 10.1111/j.1365-2982.2011.01796.x 21988661 PMC3413724

[B6] Blackshaw L. A. Page A. J. Partosoedarso E. R. (2000). Acute effects of capsaicin on gastrointestinal vagal afferents. Neuroscience 96, 407–416. 10.1016/s0306-4522(99)00547-3 10683581

[B7] Bohórquez D. V. Shahid R. A. Erdmann A. Kreger A. M. Wang Y. Calakos N.. (2015). Neuroepithelial circuit formed by innervation of sensory enteroendocrine cells. J. Clin. Invest. 125, 782–786. 10.1172/JCI78361 25555217 PMC4319442

[B8] Bravo J. A. Forsythe P. Chew M. V. Escaravage E. Savignac H. M. Dinan T. G.. (2011). Ingestion of Lactobacillus strain regulates emotional behavior and central GABA receptor expression in a mouse *via* the vagus nerve. Proc. Natl. Acad. Sci. U S A 108, 16050–16055. 10.1073/pnas.1102999108 21876150 PMC3179073

[B9] Buckley M. M. O’Malley D. (2018). Development of an *ex vivo* method for multi-unit recording of microbiota-colonic-neural signaling in real time. Front. Neurosci. 12:112. 10.3389/fnins.2018.00112 29535604 PMC5835233

[B10] Cani P. D. Joly E. Horsmans Y. Delzenne N. M. (2006). Oligofructose promotes satiety in healthy human: a pilot study. Eur. J. Clin. Nutr. 60, 567–572. 10.1038/sj.ejcn.1602350 16340949

[B11] Chimerel C. Emery E. Summers D. K. Keyser U. Gribble F. M. Reimann F. (2014). Bacterial metabolite indole modulates incretin secretion from intestinal enteroendocrine L cells. Cell Rep. 9, 1202–1208. 10.1016/j.celrep.2014.10.032 25456122 PMC4308618

[B12] Christmas D. M. Badawy A. A. Hince D. Davies S. J. Probert C. Creed T.. (2010). Increased serum free tryptophan in patients with diarrhea-predominant irritable bowel syndrome. Nutr. Res. 30, 678–688. 10.1016/j.nutres.2010.09.009 21056283

[B13] Dinan T. G. Cryan J. F. (2013). Melancholic microbes: a link between gut microbiota and depression? Neurogastroenterol. Motil. 25, 713–719. 10.1111/nmo.12198 23910373

[B14] Dinan T. G. Cryan J. F. (2017). Gut instincts: microbiota as a key regulator of brain development, ageing and neurodegeneration. J. Physiol. 595, 489–503. 10.1113/JP273106 27641441 PMC5233671

[B15] Duca F. A. Sakar Y. Covasa M. (2013). Combination of obesity and high-fat feeding diminishes sensitivity to GLP-1R agonist exendin-4. Diabetes 62, 2410–2415. 10.2337/db12-1204 23423571 PMC3712059

[B16] Ekblad E. Winther C. Ekman R. Håkanson R. Sundler F. (1987). Projections of peptide-containing neurons in rat small intestine. Neuroscience 20, 169–188. 10.1016/0306-4522(87)90010-8 2436086

[B17] Gareau M. G. Jury J. Perdue M. H. (2007). Neonatal maternal separation of rat pups results in abnormal cholinergic regulation of epithelial permeability. Am. J. Physiol. Gastrointest. Liver Physiol. 293, G198–G203. 10.1152/ajpgi.00392.2006 17510196

[B18] Gibson G. R. Roberfroid M. B. (1995). Dietary modulation of the human colonic microbiota: introducing the concept of prebiotics. J. Nutr. 125, 1401–1412. 10.1093/jn/125.6.1401 7782892

[B19] Gribble F. M. Reimann F. (2016). Enteroendocrine cells: chemosensors in the intestinal epithelium. Annu. Rev. Physiol. 78, 277–299. 10.1146/annurev-physiol-021115-105439 26442437

[B20] Grigoryan M. Kedees M. H. Charron M. J. Guz Y. Teitelman G. (2012). Regulation of mouse intestinal L cell progenitors proliferation by the glucagon family of peptides. Endocrinology 153, 3076–3088. 10.1210/en.2012-1120 22569789 PMC3380309

[B21] Hansen C. F. Vrang N. Sangild P. T. Jelsing J. (2013). Novel insight into the distribution of L-cells in the rat intestinal tract. Am. J. Transl. Res. 5, 347–358. 23634245 PMC3633977

[B22] Harada K. Sada S. Sakaguchi H. Takizawa M. Ishida R. Tsuboi T. (2018). Bacterial metabolite S-equol modulates glucagon-like peptide-1 secretion from enteroendocrine L cell line GLUTag cells *via* actin polymerization. Biochem. Biophys. Res. Commun. 501, 1009–1015. 10.1016/j.bbrc.2018.05.100 29777703

[B23] Hellström P. M. Naslund E. Edholm T. Schmidt P. T. Kristensen J. Theodorsson E.. (2008). GLP-1 suppresses gastrointestinal motility and inhibits the migrating motor complex in healthy subjects and patients with irritable bowel syndrome. Neurogastroenterol. Motil. 20, 649–659. 10.1111/j.1365-2982.2007.01079.x 18298441

[B24] Jaglin M. Rhimi M. Philippe C. Pons N. Bruneau A. Goustard B.. (2018). Indole, a signaling molecule produced by the gut microbiota, negatively impacts emotional behaviors in rats. Front. Neurosci. 12:216. 10.3389/fnins.2018.00216 29686603 PMC5900047

[B25] Jones B. Buenaventura T. Kanda N. Chabosseau P. Owen B. M. Scott R.. (2018). Targeting GLP-1 receptor trafficking to improve agonist efficacy. Nat. Commun. 9:1602. 10.1038/s41467-018-03941-2 29686402 PMC5913239

[B26] Kaelberer M. M. Buchanan K. L. Klein M. E. Barth B. B. Montoya M. M. Shen X.. (2018). A gut-brain neural circuit for nutrient sensory transduction. Science 361:eaat5236. 10.1126/science.aat5236 30237325 PMC6417812

[B27] Kakei M. Yada T. Nakagawa A. Nakabayashi H. (2002). Glucagon-like peptide-1 evokes action potentials and increases cytosolic Ca^2+^ in rat nodose ganglion neurons. Auton. Neurosci. 102, 39–44. 10.1016/s1566-0702(02)00182-0 12492134

[B28] Keast J. R. Furness J. B. Costa M. (1984). Origins of peptide and norepinephrine nerves in the mucosa of the guinea pig small intestine. Gastroenterology 86, 637–644. 10.1016/s0016-5085(84)80111-0 6199254

[B29] Kedees M. H. Guz Y. Grigoryan M. Teitelman G. (2013). Functional activity of murine intestinal mucosal cells is regulated by the glucagon-like peptide-1 receptor. Peptides 48, 36–44. 10.1016/j.peptides.2013.07.022 23927844

[B30] Kissow H. Hartmann B. Holst J. J. Poulsen S. S. (2013). Glucagon-like peptide-1 as a treatment for chemotherapy-induced mucositis. Gut 62, 1724–1733. 10.1136/gutjnl-2012-303280 23086829

[B31] Kuhre R. E. Frost C. R. Svendsen B. Holst J. J. (2015). Molecular mechanisms of glucose-stimulated GLP-1 secretion from perfused rat small intestine. Diabetes 64, 370–382. 10.2337/db14-0807 25157092

[B32] Ma X. Bruning J. Ashcroft F. M. (2007). Glucagon-like peptide 1 stimulates hypothalamic proopiomelanocortin neurons. J. Neurosci. 27, 7125–7129. 10.1523/JNEUROSCI.1025-07.2007 17611265 PMC3868967

[B33] Mao Y. K. Kasper D. L. Wang B. Forsythe P. Bienenstock J. Kunze W. A. (2013). Bacteroides fragilis polysaccharide A is necessary and sufficient for acute activation of intestinal sensory neurons. Nat. Commun. 4:1465. 10.1038/ncomms2478 23403566

[B34] McVey Neufeld K. A. Mao Y. K. Bienenstock J. Foster J. A. Kunze W. A. (2013). The microbiome is essential for normal gut intrinsic primary afferent neuron excitability in the mouse. Neurogastroenterol. Motil. 25:183–e88. 10.1111/nmo.12049 23181420

[B35] Nakagawa A. Satake H. Nakabayashi H. Nishizawa M. Furuya K. Nakano S.. (2004). Receptor gene expression of glucagon-like peptide-1, but not glucose-dependent insulinotropic polypeptide, in rat nodose ganglion cells. Auton. Neurosci. 110, 36–43. 10.1016/j.autneu.2003.11.001 14766323

[B36] O’Brien R. Buckley M. M. Kelliher A. O’Malley D. (2019). PI 3-kinase- and ERK-MAPK-dependent mechanisms underlie Glucagon-Like Peptide-1-mediated activation of Sprague Dawley colonic myenteric neurons. Neurogastroenterol. Motil. 31:e13631. 10.1111/nmo.13631 31121089

[B38] O’Malley D. Dinan T. G. Cryan J. F. (2011a). Altered expression and secretion of colonic interleukin-6 in a stress-sensitive animal model of brain-gut axis dysfunction. J. Neuroimmunol. 235, 48–55. 10.1016/j.jneuroim.2011.04.003 21565410

[B37] O’Malley D. Liston M. Hyland N. P. Dinan T. G. Cryan J. F. (2011b). Colonic soluble mediators from the maternal separation model of irritable bowel syndrome activate submucosal neurons *via* an interleukin-6-dependent mechanism. Am. J. Physiol. Gastrointest. Liver Physiol. 300, G241–G252. 10.1152/ajpgi.00385.2010 21109592

[B39] Pérez-Berezo T. Pujo J. Martin P. Le Faouder P. Galano J. M. Guy A.. (2017). Identification of an analgesic lipopeptide produced by the probiotic Escherichia coli strain Nissle 1917. Nat. Commun. 8:1314. 10.1038/s41467-017-01403-9 29101366 PMC5670229

[B41] Perez-Burgos A. Mao Y. K. Bienenstock J. Kunze W. A. (2014). The gut-brain axis rewired: adding a functional vagal nicotinic “sensory synapse”. FASEB J. 28, 3064–3074. 10.1096/fj.13-245282 24719355

[B40] Perez-Burgos A. Wang B. Mao Y. K. Mistry B. McVey Neufeld K. A. Bienenstock J.. (2013). Psychoactive bacteria Lactobacillus rhamnosus (JB-1) elicits rapid frequency facilitation in vagal afferents. Am. J. Physiol. Gastrointest. Liver Physiol. 304, G211–G220. 10.1152/ajpgi.00128.2012 23139216

[B42] Pusceddu M. M. Barboza M. Keogh C. E. Schneider M. Stokes P. Sladek J. A.. (2019). Nod-like receptors are critical for gut-brain axis signalling in mice. J. Physiol. 597, 5777–5797. 10.1113/JP278640 31652348 PMC6911019

[B43] Reimann F. Habib A. M. Tolhurst G. Parker H. E. Rogers G. J. Gribble F. M. (2008). Glucose sensing in L cells: a primary cell study. Cell Metab. 8, 532–539. 10.1016/j.cmet.2008.11.002 19041768 PMC2697331

[B44] Richards W. Hillsley K. Eastwood C. Grundy D. (1996). Sensitivity of vagal mucosal afferents to cholecystokinin and its role in afferent signal transduction in the rat. J. Physiol. 497, 473–481. 10.1113/jphysiol.1996.sp021781 8961188 PMC1160997

[B45] Riley T. P. Neal-McKinney J. M. Buelow D. R. Konkel M. E. Simasko S. M. (2013). Capsaicin-sensitive vagal afferent neurons contribute to the detection of pathogenic bacterial colonization in the gut. J. Neuroimmunol. 257, 36–45. 10.1016/j.jneuroim.2013.01.009 23481698 PMC4188534

[B46] Ryan P. M. Patterson E. Kent R. M. Stack H. O’Connor P. M. Murphy K.. (2017). Recombinant incretin-secreting microbe improves metabolic dysfunction in high-fat diet fed rodents. Sci. Rep. 7:13523. 10.1038/s41598-017-14010-x 29051554 PMC5648875

[B47] Schirra J. Nicolaus M. Roggel R. Katschinski M. Storr M. Woerle H. J.. (2006). Endogenous glucagon-like peptide 1 controls endocrine pancreatic secretion and antro-pyloro-duodenal motility in humans. Gut 55, 243–251. 10.1136/gut.2004.059741 15985560 PMC1856508

[B48] Stilling R. M. Dinan T. G. Cryan J. F. (2016). The brain’s Geppetto-microbes as puppeteers of neural function and behaviour? J. Neurovirol. 22, 14–21. 10.1007/s13365-015-0355-x 26047662

[B49] Sun E. W. de Fontgalland D. Rabbitt P. Hollington P. Sposato L. Due S. L.. (2017). Mechanisms controlling glucose-induced GLP-1 secretion in human small intestine. Diabetes 66, 2144–2149. 10.2337/db17-0058 28385801 PMC5860185

[B50] Tolhurst G. Heffron H. Lam Y. S. Parker H. E. Habib A. M. Diakogiannaki E.. (2012). Short-chain fatty acids stimulate glucagon-like peptide-1 secretion *via* the G-protein-coupled receptor FFAR2. Diabetes 61, 364–371. 10.2337/db11-1019 22190648 PMC3266401

[B51] Wichmann A. Allahyar A. Greiner T. U. Plovier H. Lunden G. Ö. Larsson T.. (2013). Microbial modulation of energy availability in the colon regulates intestinal transit. Cell Host Microbe. 14, 582–590. 10.1016/j.chom.2013.09.012 24237703

[B52] Yano J. M. Yu K. Donaldson G. P. Shastri G. G. Ann P. Ma L.. (2015). Indigenous bacteria from the gut microbiota regulate host serotonin biosynthesis. Cell 161, 264–276. 10.1016/j.cell.2015.02.047 25860609 PMC4393509

